# Prediction of Overall Survival Rate in Patients With Hepatocellular Carcinoma Using an Integrated Model Based on Autophagy Gene Marker

**DOI:** 10.3389/fgene.2021.647309

**Published:** 2021-04-01

**Authors:** Shuaiqun Wang, Dalu Yang, Wei Kong

**Affiliations:** College of Information Engineering, Shanghai Maritime University, Shanghai, China

**Keywords:** hepatocellular carcinoma, autophagy gene, survival rate, survival model, TCGA database, ICGC database

## Abstract

The autophagy cell, which can inhibit the formation of tumor in the early stage and can promote the development of tumor in the late stage, plays an important role in the development of tumor. Therefore, it has potential significance to explore the influence of autophagy-related genes (AAGs) on the prognosis of hepatocellular carcinoma (HCC). The differentially expressed AAGs are selected from HCC gene expression profile data and clinical data downloaded from the TCGA database, and human autophagy database (HADB). The role of AAGs in HCC is elucidated by GO functional annotation and KEGG pathway enrichment analysis. Combining with clinical data, we selected age, gender, grade, stage, T state, M state, and N state as Cox model indexes to construct the multivariate Cox model and survival curve of Kaplan Meier (KM) was drawn to estimate patients’ survival between high- and low-risk groups. Through an ROC curve drawn by univariate and multivariate Cox regression analysis, we found that seven genes with high expression levels, including HSP90AB1, SQSTM1, RHEB, HDAC1, ATIC, HSPB8, and BIRC5 were associated with poor prognosis of HCC patients. Then the ICGC database is used to verify the reliability and robustness of the model. Therefore, the prognosis model of HCC constructed by autophagy genes might effectively predict the overall survival rate and help to find the best personalized targeted therapy of patients with HCC, which can provide better prognosis for patients.

## Introduction

Hepatocellular carcinoma (HCC) is the most common primary liver cancer accounting for more than 95%. The main cause of HCC is chronic infection of hepatitis B virus (HBV) and hepatitis C virus (HCV) (Orcutt and Anaya, 2018). Because of the poor prognosis for HCC, the mortality of HCC has been increasing in the past few decades. The 3-year survival rate is only 12.7%, and the median survival time is only 9 months (Kim et al., 2017). Therefore, it is very important to explore the early diagnosis and accurate prognosis of liver cancer.

At present, autophagy has been proved to be associated with a variety of human diseases from neurodegenerative diseases to cancer, including HCC. Autophagy is a process in which damaged, denatured, or aged proteins and organelles are transported to lysosomes for digestion and degradation (Janku et al., 2011). Autophagy can prevent the accumulation of damaged proteins and organelles and inhibit the carcinogenesis of cells. However, once a tumor is formed, autophagy provides more abundant nutrition for cancer cells and promotes the growth of tumors (Mizushima, 2007). Acute spinal cord injury (ASCI) is considered to be a serious damage to the central nervous system. At present, the research in spinal surgery and neurology has highlighted this complexity, in which autophagy is considered to play a crucial role. Xu et al. used computational tools and published data to identify genes and molecular pathways related to ASCI and autophagy, and to identify drugs targeting genes related to ASCI and autophagy. A total of 20 potential genes, 6 important pathways, and 28 drug candidates were identified, which laid a foundation for the development of new clinical trials and new targeted therapies (Xu and Zhao, 2021). Xu et al. (2020) used a machine-learning method to analyze a large number of colon cancer patients’ data, constructed a prognosis evaluation model of colon cancer patients based on autophagy genes, and identified eight autophagy genes (CTSD, ULK3, CDKN2A, NRG1, ATG4B, ULK1, DAPK1, and SERPINA1) as key prognostic genes. Eight genes were verified to be closely related to the autophagy process of tumorigenesis and development. Wang et al. (2020) and Zhang et al. (2020) also explored the potential value of autophagy-related genes (AAGs) in lung adenocarcinoma through the autophagy gene model. Some prognosis-related autophagy genes were identified to be associated with Lung adenocarcinoma (LUAD), which provided important value for the prognosis of LUAD. In the research and treatment of laryngeal cancer, autophagy has also attracted much attention. Luo et al. (2020) established an autophagy-related model to predict the prognosis of patients with laryngeal cancer. Four key genes (IKBKB, ST13, TSC2, and map2k7) were screened out through the model, and the comprehensive analysis of ARGs expression profile and clinical results finally determined that there was a significant correlation with overall survival of laryngeal cancer. However, due to the existence of single cohort false positive rate, the results are not reliable. Therefore, in this study, we combined autophagy genes with the gene data set of HCC patients to identify the biomarkers of HCC.

Preclinical models and clinical trials have confirmed that the complex interaction between autophagy and apoptosis determines the degree of hepatocyte apoptosis and the progress of liver diseases (David, 2012). Autophagy has a great influence on liver diseases, such as alcoholic fatty liver disease, hepatitis, and fatty liver. Therefore, further study on the relationship between autophagy and tumor, and the construction of prognosis model, may be of great value for tumor treatment strategy.

In this paper, we first combined a complete set of human autophagy genes with the expression profile data of HCC and mined the autophagy genes related to prognosis. In order to explore the potential value of autophagy in HCC, the prognosis risk model of autophagy genes was constructed, and the risk value of patients was calculated. In the experiment, 62 differentially expressed autophagy genes were selected from HCC samples in the TCGA database. The autophagy genes combined with survival time to determine whether the differentially expressed autophagy genes in HCC patients are related to the overall survival time. Then the prognosis model was established, and the accuracy of the model was determined by Kaplan Meier (KM) curve and Receiver Operating Characteristic (ROC) curve. In addition, we downloaded HCC data from the ICGC database as compared group to verify the reliability and robustness of autophagy gene prognosis model.

## Data and Methods

### Data Source

Two hundred thirty-three human autophagy genes are downloaded from human autophagy gene database^1^, and HCC sample data including 50 normal samples and 374 cancer samples are downloaded from TCGA database^2^. Human genome annotation is downloaded from ENSEMBL databas^3^. Then another HCC sample data is downloaded from ICGC database^4^, which contains 202 normal samples and 243 cancer samples.

### Select Differentially Expressed Autophagy Genes

Two hundred thirty-three autophagy genes are screened from the experimental group tumor data downloaded from TCGA database, and the control group data is downloaded from the ICGC database.

The logarithm of log 2 between tumor samples and normal samples was taken to normalize the gene expression values. When the screening threshold was set as | log (foldchange) | > 1 and False Discovery Rate (FDR) < 0.05, the *p*-value was corrected by FDR. The smaller the *p*-value, the greater the difference of genes. Wilcoxon signed rank test was used to identify autophagy genes differentially expressed between tumor samples and normal samples. Finally, 62 AAGs were identified in the experimental group and 28 autophagy related genes in the control group.

### Construct Prognosis-Model and Verify Accuracy

Cox regression model is a semi-parametric regression model proposed by the British statistician D.R. Cox. The Cox model takes survival outcome and survival time as dependent variables, which can simultaneously analyze the influence of many factors on survival time. The basic form of the model is as follows:

(1) $$\text{h}\,\left(t,X\right)=h_{0}\,\left(t\right)\,\exp\left(\beta_{1}\,X_{1}+\beta_{2}\,X_{2}+\ldots +\beta_{m}\,X_{m}\right)$$

β_1,_ β_2_,……, β_m_ are the partial regression coefficient of the independent variable, which is the parameter to be estimated from the sample data; h_0_(t) is the baseline risk rate of h(t, X) when x vector is 0, which is the quantity to be estimated from the sample data.

Therefore, in this study, we used univariate and multivariate Cox regression analysis to study the correlation between overall survival and gene expression level. We used univariate Cox regression analysis to determine overall survival-related genes and narrow the range of HCC marker genes. Then we used the multivariate Cox model to construct a prognostic-related model of HCC. Seven factors including age, gender, cell grade, cell stage, T status, M status, and N status are selected as indicators of the Cox model, which is defined as follows:

(2) $$r\,i\,s\,k\,S\,c\,o\,r\,e=\sum_{i=1}^{n}x_{i}\,y_{i}$$

Where x_i_ represents the expression of gene I, y_i_ represents the Cox regression coefficient of gene I, and n represents the number of independent indicators. According to the formula of multivariate Cox model, we can calculate the median value of risk value with each patient, which can be used to divide patients into high-risk group and low-risk group. The KM curve is drawn to judge whether it exists difference in survival between two groups. Then we draw the multi-index characteristic curve (ROC), and the area under the curve indicates whether the model is meaningful.

### Enrichment Analysis

Go enrichment analysis can be divided into three parts: biological process (BP), molecular function (MC), and cell component (CC). BP is a process that is composed of orderly MCs and has many steps. MC describes the activity of an individual molecular biological. What kind of gene or organelle is located in CC.

In order to study the tumor molecular mechanism in AAGs, R packages with DOSE, CLUSTERPROFILER, ENRICHLOT, GGPLOT2, and ORG.HS.EG.DB is used to select the differential genes between the experimental group and the control group. Then we calculate the hypergeometric distribution relationship between differential genes and a specific branch in GO classification. *P*-value is obtained by Go analysis for each differential gene, and the smaller the *p*-value, the more significant the differential gene enrichment (*P* < 0.05).

The above-mentioned R-package is also used to process the two groups of data in KEGG pathway analysis. The differential genes are selected based on *P* < 0.05 and are regarded as a whole network with expression information process.

### Data Processing

In this paper, all data processing and chart analysis are based on R language (R 4.0.2) and Perl language. The limma package is used to obtain the mean value of genes with multiple rows, and the Wilcox test is utilized to take differentially expressed autophagy genes. Log2 is used to normalize the gene expression data, and the genes with *P* < 0.05 are selected as significant difference genes. In order to represent the survival difference between the two groups, the KM curve is drawn, and the area under the ROC curve is used to evaluate the performance of the model.

## Results

**FIGURE 1:**
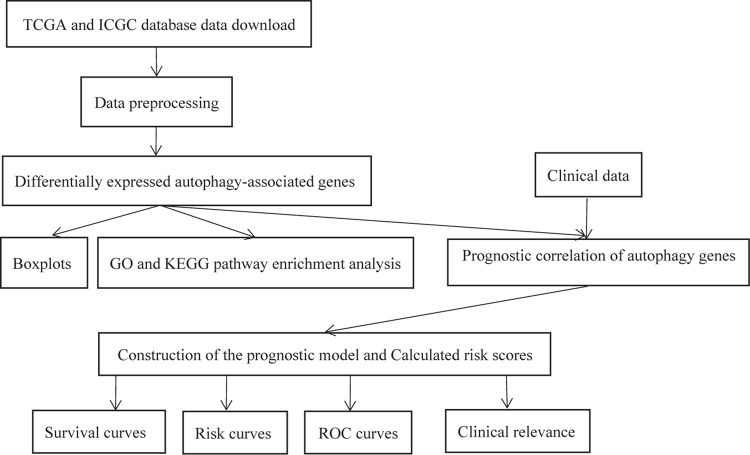
The flow chart of the experiment.

The experimental flow chart of this paper is shown in Figure 1, and we will discuss each part in detail later.

### Differential Expression of Autophagy-Related Genes

We process the data with 424 cases in the experimental group and 445 cases in the control group, respectively. In the experimental group, 50 cases are normal samples, and 374 cases are cancer samples. There were 202 normal samples, and 243 tumor samples in the control group. The Wilcox test is used to select differentially expressed autophagy genes. The screening threshold of the differential expression with 233 autophagy genes is set as | log(foldchange) | > 1 and fdr < 0.05 in the experiment group. The threshold of upregulated genes is set as log (foldchange) > 0; otherwise, it is downregulated genes. Finally, 62 autophagy-related genes that contained 4 downregulated genes and 58 upregulated genes are identified. In the control group, 28 autophagy-related genes are identified in which 2 genes are downregulated and 26 genes are upregulated.

**FIGURE 2:**
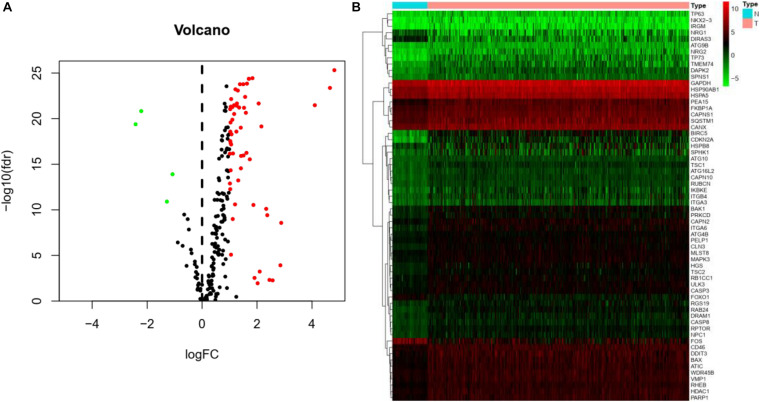
Differential expression of autophagy-related genes between normal and tumor samples in HCC. **(A)** Volcano map indicates the differential expression of 232 autophagy genes. The abscissa of volcano map is logFC and the ordinate is -log10(fdr). Black dots represent genes that have no difference between normal samples and tumor samples, while green dots and red dots indicate low and high gene expression in tumor samples, respectively. **(B)** Thermography intuitively shows the hierarchical clustering of differentially expressed autophagy-related genes in normal and tumor samples. The abscissa of the thermogram is the sample and the ordinate is the differential autophagy-related genes, the blue is the normal sample, and the pink is the tumor sample. Green, black, and red represent the level of gene expression.

**FIGURE 3:**
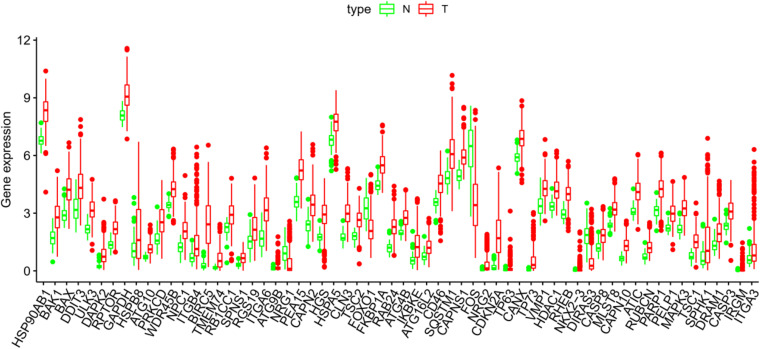
The abscissa of the box diagram represents the differential genes, and the ordinate represents the expression level of the genes. Green line represents normal samples, and red line represents tumor samples.

The Pyrogram and volcano figure of 62 autophagy-related genes in normal and tumor samples are drawn using pheatmap R package, as shown in Figure 2. In order to show the gene expression of autophagy differential genes in normal samples and tumor samples more directly, the box diagram of autophagy-related genes is also drawn using ggpubr R package, as shown in Figure 3.

### Go Enrichment and KEGG Pathway Analysis

In order to explore the molecular mechanism of autophagy-related genes in HCC, we performed go enrichment analysis and KEGG pathway analysis on sample data.

**FIGURE 4:**
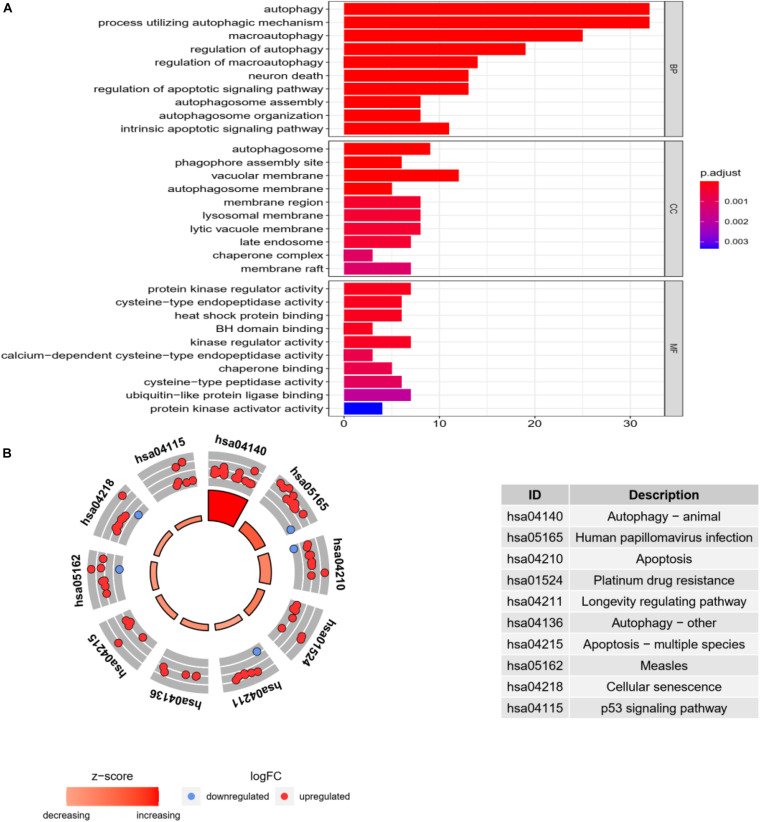
Go enrichment analysis and KEGG pathway analysis. **(A)** Go enrichment analysis histogram. From blue to red, the higher the value of *P*. adjust and the longer the length, the more genes are enriched in the pathway. **(B)** Cycle diagram of KEGG pathway analysis. The inner circle represents the Z-score value, and the larger the value, the redder the color. The outer ring represents that pathway contains the most upregulated and downregulated genes. The red dot indicates the upregulated gene and the blue dot indicates the downregulated gene. The right figure lists KEGG pathway.

In go enrichment analysis and KEGG pathway analysis, the filter condition is set as *p*. adjusted <0.05. If the pathway with *p*. adjusted does not exist, *p* < 0.05 can also be used as the selected condition. We select the top 10 process of three modules in go enrichment analysis. In BP, they are autophagy, process utilizing autophagy mechanism, macroautophagy, regulation of autophagy, regulation of macroautophagy, neuron death, regulation of apoptotic signaling pathway, autophagosome assembly, autophagosome organization, and intrinsic apoptotic signaling pathway. The top 10 process of BP is basically related to the activity of autophagy. The top 10 process of CC is related to the activity of organelles but also includes autophagosome. The top 10 process of MF is basically the activity of individual molecular biology, such as activated protein kinase regulator activity and cysteine-type endopeptidase activity. They are shown as in Figure 4A.

In KEGG pathway analysis, the top 10 pathways with the most enriched genes are selected according to the count number of genes in the pathway, as shown in Figure 4B.

### Construct Prognosis-Model of Autophagy-Related Gene

We use all autophagy gene expression levels as input for univariate Cox regression analysis, and finally find that 62 AAGs are associated with the survival rate of HCC patients. Among them, 32 AAGs are selected as risk factors (*p* < 0.05; HRs, 1.003115658-1.041319005), and their overexpression may reduce the survival rate of patients.

By multivariate Cox regression analysis, 14 AAGs are associated with HCC patients as shown in Table 1. When the *p*-value of the gene is greater than 0.05 in multivariate analysis, we retain the gene as complement to other genes. The risk value of each gene can be calculated based on 14 AAGs with Formula (2).

**TABLE 1:** Multivariate Cox regression analysis of survival rate in patients with HCC.

**Genes**	**Coef**	**HR**	**HR.95L**	**HR.95H**	***p*-value**
SPNS1	1.617445499	5.040198664	2.08995838	12.15507582	0.00031676
ATIC	0.664084569	1.942711289	1.307840069	2.88577116	0.001004542
MLST8	–0.760676142	0.467350325	0.296519154	0.736601071	0.001049409
RHEB	0.530714439	1.700146525	1.152867022	2.50722603	0.00741324
HDAC1	0.465738387	1.593190146	1.102562075	2.302142345	0.013144435
HSPB8	0.153099368	1.165440781	1.023859491	1.326600207	0.020515825
SQSTM1	0.230890229	1.259720952	1.029756152	1.541041413	0.024763062
HSP90AB1	–0.288939881	0.749057236	0.564635872	0.993714306	0.045103224
CASP8	–0.469959004	0.625027892	0.379278762	1.030007225	0.065189281
BIRC5	0.184584243	1.202718297	0.985307028	1.468102086	0.06960683
HGS	–0.356212259	0.700323954	0.475715593	1.030980795	0.07102271
FOXO1	–0.256847744	0.773485973	0.56725571	1.054692864	0.104503719
RGS19	–0.254924427	0.774975063	0.559873529	1.072717885	0.124343514
ATG10	0.396133246	1.486067317	0.868989001	2.541339495	0.147893068

### Verify Model Performance

In order to verify the accuracy of the model, we draw KM survival curves of HCC experimental group and control group, respectively. When the value of *p* is equal to 1.987e-08 in the experimental group, the 3-year survival rate is 46.2% in the high-risk group and 73.6% in the low-risk group.

**FIGURE 5:**
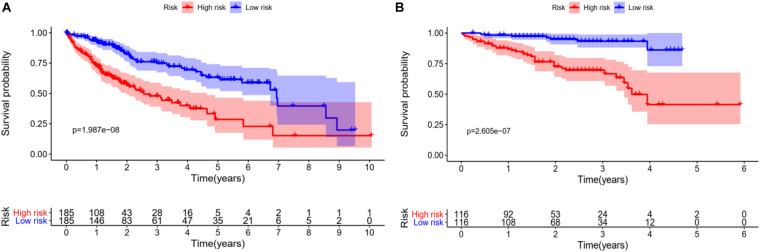
Survival analysis of HCC patients, red line represents high-risk group, blue line represents low-risk group. **(A)** KM overall survival curve of the experimental group in TCGA database shows significantly difference between high-risk group and low-risk group. **(B)** Based on the ICGC database, the KM survival curve of the control group.

In the control group, the 3-year survival rate is 66.7% in the high-risk group and 93.3% in the low-risk group with *p* equal to 2.605e-07. It can be seen that the survival rate of low-risk patients is always higher than that of high-risk groups, whether in the experimental group or the control group, as shown in Figures 5A,B.

**FIGURE 6:**
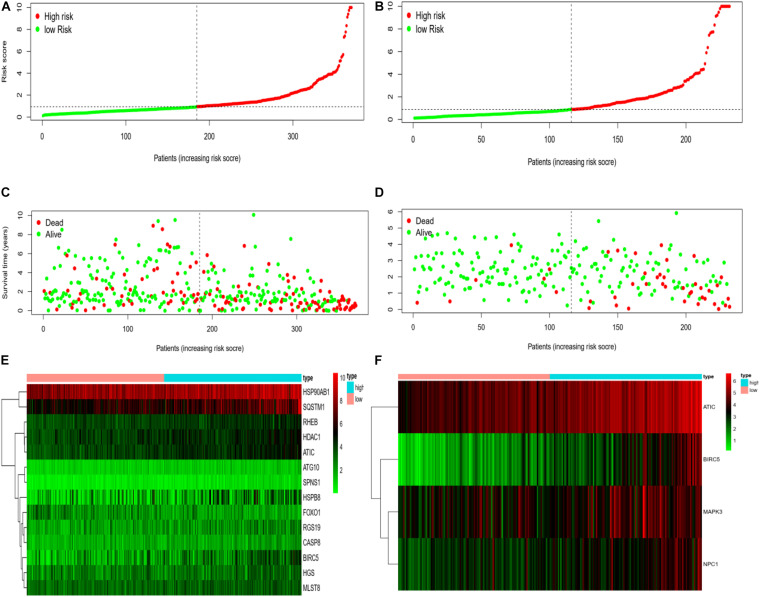
**(A)** Risk curve of HCC patients in experimental group based on TCGA database. Green line represents low risk and red line represents high risk. The dotted line is the critical value to distinguish between high and low risks. **(B)** The risk curve of HCC patients in control group based on ICGC database. **(C)** Based on TCGA database, the scatter plot of HCC patients in experimental group. The green dot represents survival, and the red dot represents mortality. With the increase of patient risk value, the number of deaths also increases. **(D)** Based on ICGC database, the scatter plot of HCC patients with different survival status in the control group. **(E)** Based on TCGA database, the calorimetry of gene expression for HCC patients with different risk values in experimental group. Red represents high-risk gene expression. **(F)** Based on the ICGC database, the calorimetry of gene expression of HCC patients with different risk values in the control group.

We rank the risk values of HCC patients from low to high, and draw a scatter diagram to analyze the survival characteristics of patients. From the scatter chart of the experimental group and the control group in Figures 6A–F, we can see that the mortality rate increases with the enlargement of the risk value. In the thermogram of the experimental group, the autophagy gene HSP90AB1 is highly expressed in the high and low risk groups, and the expressed values of the autophagy genes SQSTM1, RHEB, HDAC1, ATIC, HSPB8, and BIRC5 increase with the enlargement of risk value, which are all regard as high-risk genes of HCC. Because the data of the control group is less than that of the experimental group, four high-risk autophagy genes, namely ATIC, BIRC5, MAPK3, NPC1, are finally displayed in the thermogram.

**FIGURE 7:**
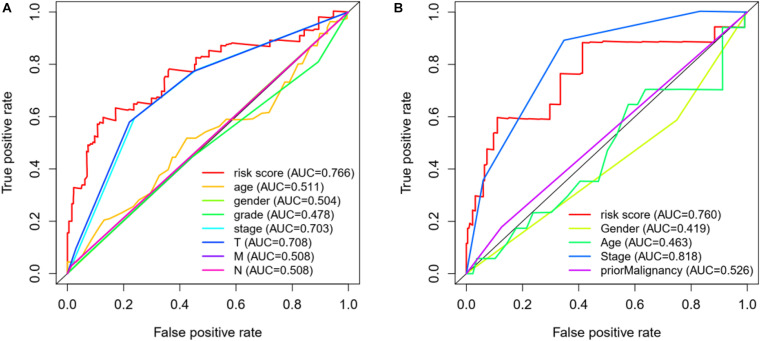
**(A)** ROC curve analysis and clinical characteristics analysis of HCC autophagy-related genes based on TCGA database. The AUC value of gene prognosis index based on autophagy related genes is higher than that of other clinical traits. **(B)** ROC curve analysis and clinical characteristics analysis of HCC autophagy-related genes based on ICGC database.

In addition, we also construct a multivariate ROC curve to evaluate the accuracy of the model. According to Figures 7A,B, the area of ROC risk curve in experimental group based on TCGA database is 0.766, which proves that the performance of prognosis index based on autophagy related genes is better than that of other clinical traits. The area of ROC risk curve is 0.760, which is better than any other index except stage. On the one hand, for Figure 7A, the ROC curve of T state is good, but for Figure 7B, T state has been filtered because the ROC curve of T state is too bad. Meanwhile, the ROC curve of stage in Figure 7A is general, and the stage ROC curve in Figure 7B is better. This phenomenon indicates that these two indicators in clinical traits are not stable and have poor robustness. On the other hand, the risk score has excellent ROC curve performance and good robustness in both groups, which may be suitable for a variety of databases.

## Discussion

HCC is a major cancer in the worldwide. Due to the slow progress of complex molecular targeted therapy and lack of effective biomarkers for the prognosis prediction of HCC (Li et al., 2020), we urgently need effective methods to find biomarkers to provide therapeutic options for patients with HCC.

Sim et al. proposed that immunotherapy can be for HCC patients. Meanwhile, immune checkpoint inhibitors have good tolerance in HCC and have significant clinical benefits. Then the researchers began to explore the potential synergy of immune checkpoint inhibitors with local and other systemic therapies. In order to the optimal use of these drugs, efforts have been made to identify the molecular biomarkers of treatment response and drug resistance (Sim and Knox, 2018). Sorafenib, a kinase inhibitor, is the only drug approved for the treatment of advanced HCC (Lovet and Hernandez-Gea, 2014). However, sorafenib has developed primary or acquired drug resistance, which has produced obstacles in the treatment of HCC. Sun et al. (2017) found that sorafenib can induce autophagy in HCC resulting in cell death or survival, which affects sorafenib treatment. Fibrolamellar HCC is a kind of primary liver tumor and is relatively rare (Kassahun, 2016). Most cases are diagnosed as the advanced stage for the initial diagnosis. Surgery (resection/liver transplantation) is the main treatment and the only potential treatment option. However, due to the high recurrence rate, the prognosis of patients is also very poor. With the development of genome sequencing, more and more potential gene biomarkers have been found and have been discovered to have predictive value for HCC patients.

In this study, we combined a complete set of human autophagy genes with HCC for the first time to explore and verify the potential value of AAGs in HCC. First, we selected 62 differentially expressed AAGs from 50 normal samples and 374 cancer samples downloaded from the TCGA database. Because these genes may be deeply involved in the initiation of HCC, we conduct GO and KEGG analysis on these genes. AAGs were mainly enriched in macroautophagy, intrinsic apoptotic signaling pathway and regulation of apoptotic signaling pathway by Functional Enrichment Analysis, which is consistent with previous studies. Autophagy is a physiological process that depends on lysosomal to degrade cytoplasmic proteins and organelles, and eliminates the response of misfolded proteins and damaged organelles to cell stress (Ryter et al., 2013). In the analysis of KEGG pathway, AAGs are mainly concentrated in apoptosis, p53 signaling pathway, and cellular senescence. There have been studies on the inhibition of proliferation and metastasis of gastric cancer (Wei and Wang, 2017), and the migration and invasion of cervical cancer cells by the p53 signaling pathway (Liu et al., 2018). Furthermore, univariate and multivariate Cox regression analysis are used to select a more useful marker and establish a risk model for predicting the prognosis of HCC. Finally, we find that seven genes with high expression levels, including HSP90AB1, SQSTM1, RHEB, HDAC1, ATIC, HSPB8, and BIRC5, are associated with poor prognosis in HCC patients. We demonstrate that these seven gene signatures are superior to conventional clinicopathological factors for HCC patients. Their ability to predict survival is also demonstrated in the ICGC database. Through the ROC curve and AUC value, it is shown that the model performs well in both the experimental group and the control group. Therefore, we believe that the risk score model based on seven genes might be used to divide HCC patients into a high-risk group and low-risk group. It can be useful for detection recurrence or early prevention in high-risk groups, and it has great significance for the treatment of the disease.

Nowadays, most of the prognostic features for cancer based on expression profiles have been proposed with the development of large-scale public databases. For example, Jia et al. (2018) mined genes with prognostic value in the glioblastoma microenvironment based on TCGA database. Xiang et al. (2020) discovered that CLDN10, a key immune-related gene, is associated with lymph node metastasis in papillary thyroid carcinoma. Due to lack of enough cases in our control group, there is a quantitative difference between the four high-expression autophagy genes screened in the control group and the seven autophagy genes in the experimental group. However, it does not affect the final prognosis analysis results.

## Conclusion

In this study, we deeply explore the function of autophagy genes in HCC and build a reliable model based on the differential expression of autophagy genes. However, our model does have some potential limitations. On the one hand, due to the lack of sufficient cases and reliable HCC cells in the control group, there will be partial bias between our control group and the experimental group. On the other hand, several other important clinical information, such as various treatments and the number of lymph nodes, is not available now. In order to provide better prognosis for patients, further prospective experiments can be used to test the clinical efficacy and help to find the best personalized targeted therapy in the future.

## Data Availability Statement

The original contributions presented in the study are included in the article/supplementary material, further inquiries can be directed to the corresponding author/s.

## Ethics Statement

Written informed consent was obtained from the individual(s) for the publication of any potentially identifiable images or data included in this article.

## Author Contributions

SW and DY participated in its design, performed all the molecular biological analyses of the data, and construct the prognosis model and analysis data. DY prepared data and made pre-processing with data. WK helped with data interpretation and manuscript drafting. All authors read and approved the final manuscript.

## Conflict of Interest

The authors declare that the research was conducted in the absence of any commercial or financial relationships that could be construed as a potential conflict of interest.

## Footnotes

1 http://www.autophagy.lu/

2 https://portal.gdc.cancer.gov/

3 http://asia.ensembl.org/index.html

4 https://dcc.icgc.org/releases/current/Projects
